# Seed-to-Seedling Transition: Novel Aspects

**DOI:** 10.3390/plants11151988

**Published:** 2022-07-30

**Authors:** Galina Smolikova, Sergei Medvedev

**Affiliations:** Department of Plant Physiology and Biochemistry, St. Petersburg State University, 199034 St. Petersburg, Russia; s.medvedev@spbu.ru

**Keywords:** seed, germination, post-germination, seedling establishment, gene expression, hormonal regulation

## Abstract

Transition from seed to seedling represents a critical stage in plants’ life cycles. This process includes three significant events in the seeds: (i) tissue hydration, (ii) the mobilization of reserve nutrients, and (iii) the activation of metabolic activity. Global metabolic rearrangements lead to the initiation of radicle growth and the resumption of vegetative development. It requires massive reprogramming of the transcriptome, proteome, metabolome, and attendant signaling pathways, resulting in the silencing of seed-maturation genes and the activation of vegetative growth genes. This Special Issue discusses the mechanisms of genetic, epigenetic, and hormonal switches during seed-to-seedling transitions. Detailed information has also been covered regarding the influence of germination features on seedling establishment.

## 1. Introduction

In higher plants’ life cycle, transitions from seed to seedling represent a critical developmental step, dramatically affecting plant ontogenesis and stress tolerance. To establish seedlings at the appropriate time, seeds have evolved mechanisms to maintain dormancy until environmental conditions (temperature, moisture, and light) are favorable for germination [1,2,3]. Three phases of seed germination are recognized [4,5,6]. Phase I is primarily a physical process characterized by rapid water uptakes and the hydration of macromolecules. It results in the resumption of mitochondrial respiration and activation of DNA and membrane reparation. During phase II, the main metabolic processes, for example, the synthesis of new RNA, lipids, and proteins as well as the mobilization of reserve nutrients are activated. Phases I and II encompass important events that drive the seed from a quiescent to a germination state. Phase III represents the post-germination stage characterized by the progressive division and elongation of cells in the embryonic axes resulting in so-called “radicle protrusion.” This ultimately results in the loss of desiccation tolerance occurring in orthodox seeds during late germination [7,8,9].

Before plants can resume vegetative development, a massive reprogramming of the transcriptome and attendant signaling pathways is required. This results in the silencing of seed maturation genes and activation of vegetative growth genes. These genes, as a rule, are methylated during seed development and demethylated during germination [10]. A network of transcription factors known as LAFLs (LEAFY COTYLEDON1 and 2, ABSCISIC INHIBITOR3, and FUSCA3) and DELAY OF GERMINATION1 (DOG1) was described as the negative regulators of seed germination and should be repressed before the initiation of seedling development [11,12]. Their repression is associated with chromatin-remodeling complexes Polycomb Repressive Complex 1 and 2 (PRC1 and PRC2), as well as the PICKLE (PKL) and PICKLE-RELATED2 (PKR2) proteins [11,13,14].

## 2. Novel Aspects Discussed in the Special Issue

Smolikova et al. [15] reviewed the epigenetic and hormonal switches regulating seed-to-seedling transitions. Abscisic acid and gibberellins act as central endogenous regulators, controlling seed dormancy and germination antagonistically. However, in recent years, other hormones such as ethylene, cytokinins, brassinosteroids, auxins, and jasmonates have also been shown to be involved. The main result of hormone activity is the suppression of genes controlling seed maturation and the activation of those involved in vegetative growth. As previously described, epigenetic signaling provides a multifactorial and robust basis for the regulation of plant development and adaptation [16,17,18,19]. Smolikova et al. [15], therefore, also described primary epigenetic mechanisms involved in seed development and transitions from seed to seedling, such as DNA methylation, posttranslational modification of histones, and interaction with non-coding RNAs.

Research conducted by Smolikova et al. [20] deals with RNA sequencing-based transcriptomics in the embryonic axes isolated from pea seeds just before and after radicle protrusion. Seed-to-seedling transition was shown to involve a loss of desiccation tolerance, the initiation of secondary metabolism, and the activation of genes involved in defense responses to biotic stress. Many genes associated with auxin, ethylene, salicylic, and jasmonic acids were upregulated, while genes involved in the synthesis of transcription factors ABI3, ABI4, ABI5, and LEA14 were downregulated. Importantly, among LAFL genes, only *ABI3* was expressed. Authors further observed the downregulation of ABA-related genes *HVA22E*, *LTI65/RD29B*, and *LTP4* involved in responses to water deprivation as well as *PER1* involved in the suppression of ABA catabolism and GA biosynthesis via reactive oxygen species elimination.

Next, Arif et al. [21] reviewed the genetic aspects of seed longevity and their relationship with seedling viability. The authors defined seed longevity as “the maximum time period during which seeds can germinate and produce viable seedlings capable of developing into healthy plants and bearing seeds for the next generation.” The authors summarized the most relevant genetic studies on seed longevity performed in Arabidopsis and some crop species such as rice, barley, wheat, maize, soybean, tobacco, lettuce, and tomato. This review contains further information about collections of various crops available worldwide and new emerging technologies for research on seed longevity.

Another three research studies, conducted by Ducatti et al., Ribeiro-Oliveira et al., and Wang et al., paid attention to the role of germination on seedling establishment [22,23,24]. Ducatti et al. [22] analyzed gene expression patterns during the germination of soybean seeds with different vigor. The authors showed a high correlation between germination and vigor for twenty genes. Among the target genes were *EXPANSIN-LIKE A1*, *XYLOGLUCAN ENDOTRANSGLUCOSYLASE/HYDROLASE 22*, *65-KDA MICROTUBULE-ASSOCIATED PROTEIN*, *XYLOGLUCAN ENDOTRANSGLUCOSYLASE/HYDROLASE 2*, and *N-GLYCOSYLASE/DNA LYASE OGG1*. These transcripts are suggested for use as a tool for evaluating seed vigor.

Ribeiro-Oliveira et al. [23] showed that acceleration of water uptake during seed germination influences seedling vigor at the post-germination stage. The authors measured water dynamics in soybean seeds during germination, in seedlings during development, and clarified their relationship. They demonstrated that water dynamics are associated with embryo and seedling vigor and, thus, can help to predict seed vigor.

Wang et al. [24] selected six grape varieties’ conditions for the most efficient seed germination and seedling development. The authors tested different seed treatments such as stratification, chemical substances, beak cutting, and pre-germination to find the optimal combination for each variety. They propose using the developed approach for characterizing the germination and post-germination growth of seeds obtained by an intraspecific hybridization of grape varieties.

## 3. Future Perspectives

Transitions from seed to seedling are associated with a complex temporal sequence of signals. Exciting prospects for studying this phenomenon lie in the avenue of the epigenetic regulation of metabolic rearrangements. Transitions between active and repressive chromatin states in germinating seeds are still under active investigation, and the underlying molecular mechanisms remain largely unknown. Our current knowledge mainly arises from studies in Arabidopsis. However, the mechanisms of seed germination and seedling establishment in another plant species may differ significantly. Comparative studies between Arabidopsis and other crops will certainly enrich practical applications. They will provide ample opportunities for dissecting the role of epigenetic variations in trait regulation, which can be utilized in crop improvement. The locus-specific manipulation of DNA methylation by epigenome-editing tools can facilitate the molecular breeding of important crop plants. We hope that the manuscripts from this Special Issue stimulate additional research on this critical topic.
